# Does self-report of multimorbidity in later life predict impaired physical functioning, and might this be useful in clinical practice?

**DOI:** 10.1007/s40520-020-01500-8

**Published:** 2020-02-13

**Authors:** Michael A. Clynes, Gregorio Bevilacqua, Karen A. Jameson, Cyrus Cooper, Elaine M. Dennison

**Affiliations:** 1 MRC Lifecourse Epidemiology Unit, University of Southampton, Southampton General Hospital, Southampton, SO16 6YD UK; 2 National Institute for Health Research Musculoskeletal Biomedical Research Unit, University of Oxford, Oxford, OX3 7LE UK; 3 Victoria University of Wellington, Wellington, New Zealand

**Keywords:** Ageing, Multimorbidity, Non-communicable diseases, Older people, Physical functioning

## Abstract

**Background:**

Multimorbidity has been shown in several studies to relate to impaired physical function in later life.

**Aims:**

To examine if self-report of multimorbidity predicts impaired physical functioning, as assessed by formal physical function testing, in community-dwelling older adults.

**Methods:**

Non-communicable diseases (NCDs) were self-reported by 443 older community-dwelling UK adults via questionnaire, asking the question: ‘Have you been told by a doctor that you have any of the following conditions?’ Assessments of walking speed, chair stands and balance allowed us to create a composite score (0–12) on which impaired physical functioning was defined as ≤ 9.

**Results:**

The mean age of participants was 75.5 ± 2.5 years for men and 75.8 ± 2.6 for women. The proportion of individuals with impaired physical functioning was 71.2% in women and 56.9% in men. Having four or more NCDs was associated with an increased risk of poor physical function in men and women (*p* < 0.05). The number of medications and medicated systems was associated with gait speed (*p* < 0.03 and < 0.02, respectively) and timed up-and-go tests (*p* < 0.03 and < 0.02, respectively) in women but not men.

**Discussion and conclusion:**

Self-report of 4 or more NCDs was associated with an increased risk of poor physical function, an outcome which has previously been associated with adverse clinical sequelae. This observation may inform development of a simple screening tool to look for poor physical function in older adults.

**Electronic supplementary material:**

The online version of this article (10.1007/s40520-020-01500-8) contains supplementary material, which is available to authorized users.

## Introduction

An increase in life-expectancy and a subsequent ageing population have led to a higher prevalence of chronic, non-communicable diseases (NCDs) [1]. These chronic diseases contribute to a substantially increased risk of morbidity and mortality in older people [2]. The number of NCDs rises substantially with age [3]; a study by Bayliss and colleagues, utilising a survey of members of a health maintenance organisation aged 65 and over, found the average person had 8.7 chronic diseases [4] while another Canadian study reported the number of chronic diseases varies from 2.8 in young patients to 6.4 among older patients [5].

Reduced physical functioning in later life has personal and societal impacts, with an increased propensity to fall, and inability to self-care [6, 7]. Physical functioning tests are widely used tools in the research setting for exploring the reduction of physical functioning in older persons, and are part of the assessment for a diagnosis of sarcopenia [8, 9]. Studies have linked poor performance in these tests with nursing home and hospital admissions [10, 11]. Furthermore, impaired physical functioning in such tests may predict falls and fracture risk [6, 7]. However, adoption in clinical practice is difficult due to time and space constraints.

Individual NCDs have been shown in several studies to relate to impaired physical functioning [3, 4, 12]. Other researchers have suggested that the number of NCDs might be useful as a screening tool for predicting reduced physical functioning in older adults [13]. However, most studies have been undertaken in populations drawn from secondary care [14, 15] or frailer elderly [16, 17].

In the current study we therefore sought to explore, in a cohort of community-dwelling older adults in the UK, whether self-report of number of NCDs, medications, and medicated systems predicted poor physical functioning in such a population. If this was the case, it might be useful to clinicians caring for older community-dwelling adults as a simple way of identifying those at risk of poor physical function and associated clinical sequelae, and lead to the development of a simple screening tool for poor physical functioning.

## Methods

Participants were recruited from the Hertfordshire Cohort Study (HCS), a population-based sample of men and women born during 1931–1939 in Hertfordshire. Subjects were visited at home by a trained nurse, who administered a lifestyle questionnaire. The visits also included measurements of height and weight to calculate body mass index (BMI) and the identification of a number of NCDs and the medications participants were taking.

NCDs were self-reported by participants via questionnaire, asking the question: ‘Have you been told by a doctor that you have any of the following conditions?’. The following NCDs were recorded: hypertension, heart disease, stroke, diabetes, lung disease, thyroid disease, rheumatoid arthritis, osteoarthritis, multiple sclerosis, vitiligo, depression, Parkinson’s disease, peripheral arterial disease, osteoporosis and cancer (Supplementary Table 2). Participants were also asked to list other significant medical conditions, but few participants had any other individualised concerns. No participants were recruited with known dementia or cognitive impairment.

Smoker status was categorised as never smoked, ex-smoker or current smoker depending on the participants’ answers to the questions “Have you ever smoked regularly?” and “Do you still smoke regularly?”. Participants were asked how often they currently drank different types of alcohol (beer, wine, spirits, etc.) and how much they normally drank each time. This was used to estimate their alcohol consumption in units per week.

Participants were asked for details of any medications they were currently taking. These were grouped according to the system medicated: cardiovascular, respiratory, gastro-intestinal, endocrine, central nervous system, malignant disease and immunosuppression, nutrition and blood, musculoskeletal and joint disease, eye, ear, nose, skin, genito-urinary tract and miscellaneous.

The physical functioning score was derived from tests of gait speed, chair rises, and balance. To test walking speed, an 8 foot course, with no obstructions for an additional foot at either end, was marked out on the floor. Participants were asked to walk at their customary pace and the time taken was recorded using a stopwatch. The use of assistive devices, such as canes, was permitted if required. Gait speed was determined by dividing the distance traversed by the time between the first and last step.

In the timed up-and-go test, participants were asked to rise from a chair, walk 3 m, turn around, walk back to the chair and sit down again. The use of mobility aids was permitted if required. Time taken was recorded using a stopwatch.

To test chair rises, participants crossed their arms across their chest and stood up. Those who could complete this task were asked to stand up and sit down again a total of five times. The time was taken from their initial sitting position until they were standing on the fifth repetition.

The tandem stands tested the participants’ ability to maintain their balance. The standing balance test involved a semi-tandem stand where participants placed one foot in front of the other such that the big toe of one foot was touching the side of the heel of the other. If participants could not hold the semi-tandem stand for 10 s, they did a side-by-side stand (standing with the feet side-by-side). If they could hold the semi-tandem stand for 10 s, they also attempted a full tandem stand where participants placed one foot in front of the other (touching heel to toe) and held this position for as long as they could up to 10 s.

For the walking test and chair stands, those who could not complete the test were given a score of 0. The remaining participants' times were divided into quartiles and given a score of 1–4, slowest to fastest quartile. For the balance test, if the participant maintained balance in the full tandem stand for 10 s, they were given a score of 4; if they obtained a time ≥ 3 and < 10 s, they scored 3; if they obtained a time of less than 3 s but were able to maintain a semi-tandem stand, they scored 2; if they could not do the semi-tandem stand but could do the side-by-side stand, they scored 1; and if they could not do either the semi-tandem or the side-by-side stand, they scored 0. The scores for the walking test, chair rises and balance test were then summed. The maximum possible score was 12 and the minimum was 0. In keeping with previous work, those with a score equal to or lower than 9 were designated as having impaired physical functioning [18, 19].

### Statistical analysis

The physical functioning outcomes were assessed for normality and transformed where necessary using the Fisher-Yates rank-based inverse normal transformation to produce z-scores. Descriptive statistics for continuous variables were expressed as mean and standard deviation (SD) or median and interquartile range (IQR) as appropriate. Categorical variables were expressed as frequency and percentage. Differences between men and women were assessed using Student’s *t* tests, Mann–Whitney tests or Pearson’s *χ*^2^ tests, as appropriate. Linear and logistic regression analyses were used to examine the associations between the number of NCDs, medications and systems medicated and physical functioning outcomes. The regression analyses were undertaken with and without adjusting for the following demographic and lifestyle confounders: age, BMI, smoker status, alcohol consumption and social class. The results of the regression analyses are presented as regression coefficients (*β*) or odds ratios (OR) and 95% confidence intervals (CI). A *p*-value of ≤ 0.05 was considered to be statistically significant. The analyses were conducted using Stata version 14.

## Results

Table 1 displays baseline characteristics of the participants. We explored the impact of number of NCDs on physical functioning (Table 2). In both sexes having up to three NCDs, did not significantly impact physical functioning as assessed by all measures, with the exception of chair rises in women where having 3 NCDs was associated with impaired chair rises (*β* 0.54, 95% CI 0.05, 1.04 *p* = 0.032). In both men and women, however, self-reported 4 or more NCDs were associated with impaired timed up-and-go (men: *β* 1.00, 95% CI 0.45, 1.56 *p* < 0.01; women: *β* 0.90, 95% CI 0.46, 1.34 *p* < 0.01), chair rises (men: β 1.43, 95% CI 0.82, 2.03 *p* < 0.01; women: β 0.99, 95% CI 0.48, 1.51 *p* < 0.01) and physical functioning score (men: β − 1.01, 95% CI − 1.64, − 0.37 *p* < 0.01; women: β − 0.59, 95% CI − 1.12, − 0.06 *p* = 0.029). Having 4 or more NCDs was also associated with reduced gait speed in women (β − 0.46, 95% CI − 0.91, − 0.01 *p* = 0.047) and impaired tandem stand in men (OR 5.49, 95% CI 1.43, 21.01 *p* = 0.013).

**Table 1:** Baseline characteristics of study participants, physical functioning, NCDs, medications and medicated systems, by sex

Participants’ characteristics	Men			Women			*p*-value
	*N*	Mean	SD	*N*	Mean	SD	
Age (years)	222	75.5	2.5	221	75.8	2.6	0.280
Height (cm)	221	172.7	6.5	217	158.8	6.1	< 0.001

	*N*	Median	IQR	*N*	Median	IQR	
Weight (kg)	221	81.7	74.5–89.4	221	70.0	62.2–79.1	< 0.001
BMI (kg/m^2^)	221	27.4	25.3–29.9	217	27.5	24.6–31.6	0.409
Activity time in last 2 weeks (min/day)	205	176	105–270	210	200	135–283	0.089
Alcohol consumption (units per week)	222	6.5	1.0–14.0	221	0.5	0.0–3.5	< 0.001

	Total *N*	*N*	%	Total *N*	*N*	%	
Smoker status	222			221			< 0.001
Never smoked		85	38.3		142	64.3	
Ex-smoker		126	56.8		73	33.0	
Current smoker		11	5.0		6	2.7	
Social class	211			221			0.520
I–IIINM		89	42.2		100	45.2	
IIIM–V		122	57.8		121	54.8	

Physical functioning							
	*N*	Mean	SD	*N*	Mean	SD	
Gait speed (m/s)	207	0.78	0.17		0.73	0.18	0.006

	Total N	*N*	%		*N*	%	
Tandem stand < 10 s	214	43	20.1	213	56	26.3	0.129

	*N*	Median	IQR	*N*	Median	IQR	
6 m timed up-and-go (sec)	203	11.2	10.0–13.1	203	11.8	10.0–14.0	0.138
Chair rises (sec)	196	15.8	13.6–18.8	187	17	13.8–20.2	0.049
Physical functioning score	204	9.0	7.0–11.0	198	8.0	7.0–10.0	0.003

	Total *N*	*N*	%	Total *N*	*N*	%	
Low physical functioning score (< = 9)	204	116	56.9	198	141	71.2	0.003

Self-reported NCDs							
	Total *N*	*N*	%	Total *N*	*N*	%	
Hypertension	222	109	49.1	221	101	45.7	0.474
Heart disease	222	55	24.8	221	34	15.4	0.014
Stroke	222	13	5.9	221	12	5.4	0.846
Diabetes	222	36	16.2	221	24	10.9	0.099
Lung disease	222	30	13.5	221	31	14.0	0.875
Thyroid disease	222	14	6.3	221	29	13.1	0.015
Rheumatoid arthritis	222	10	4.5	221	11	5.0	0.815
Osteoarthritis	222	75	33.8	221	96	43.4	0.037

NCDs, medications, and medicated systems							
	*N*	Median	IQR	*N*	Median	IQR	
Number of NCDs	222	1	1.0–2.0	221	1	0.0–2.0	0.939
Number of medications	222	4.5	2.0–6.0	221	4	2.0–7.0	0.799
Number of systems medicated	222	2	1.0–4.0	221	3	2.0–4.0	0.079

	Total *N*	*N*	%	Total *N*	*N*	%	
Number of NCDs	222			221			0.125
0		41	18.5		56	25.3	
1		80	36.0		61	27.6	
2		61	27.5		52	23.5	
3		21	9.5		26	11.8	
4 or more		19	8.6		26	11.8	
Number of medications	222			221			0.384
0		17	7.7		13	5.9	
1–2		42	18.9		53	24.0	
3–5		84	37.8		71	32.1	
6 or more		79	35.6		84	38.0	
Number of systems medicated	222			221			0.484
0		17	7.7		13	5.9	
1		50	22.5		41	18.6	
2		56	25.2		50	22.6	
3		36	16.2		46	20.8	
4 or more		63	28.4		71	32.1

**Table 2:** Number of NCDs (categorical) as an explanatory variable for physical functioning outcomes, by sex

	Men				Women			
	*N*	Regression coefficient	95% CI	*p*-value	*N*	Regression coefficient	95% CI	*p*-value
Gait speed (FY *z*-score)	196				202			
1 NCDs		− 0.01	(− 0.38, 0.36)	0.968		− 0.14	(− 0.49, 0.20)	0.408
2 NCDs		0.17	(− 0.23, 0.57)	0.397		− 0.26	(− 0.62, 0.10)	0.15
3 NCDs		0.05	(− 0.50, 0.59)	0.870		− 0.21	(− 0.68, 0.27)	0.395
4 or more NCDs		− 0.54	(− 1.11, 0.04)	0.068		− 0.46	(− 0.91, − 0.01)	0.047
Timed up-and-go (FY *z*-score)	192				200			
1 NCDs		− 0.03	(− 0.36, 0.31)	0.869		0.13	(− 0.20, 0.46)	0.453
2 NCDs		− 0.09	(− 0.46, 0.27)	0.606		0.20	(− 0.14, 0.54)	0.242
3 NCDs		0.47	(− 0.03, 0.98)	0.066		0.28	(− 0.17, 0.73)	0.225
4 or more NCDs		1.00	(0.45, 1.56)	< 0.001		0.90	(0.46, 1.34)	< 0.001
Chair rises (FY *z*-score)	185				185			
1 NCDs		0.02	(− 0.34, 0.38)	0.916		0.00	(− 0.36, 0.36)	0.981
2 NCDs		0.18	(− 0.21, 0.56)	0.361		0.07	(− 0.32, 0.46)	0.713
3 NCDs		0.49	(− 0.05, 1.04)	0.077		0.54	(0.05, 1.04)	0.032
4 or more NCDs		1.43	(0.82, 2.03)	< 0.001		0.99	(0.48, 1.51)	< 0.001
Physical functioning score (FY *z*-score)	194				194			
1 NCDs		0.03	(− 0.37, 0.43)	0.889		− 0.11	(− 0.51, 0.28)	0.563
2 NCDs		0.11	(− 0.32, 0.54)	0.623		− 0.33	(− 0.74, 0.08)	0.115
3 NCDs		− 0.30	(− 0.89, 0.29)	0.320		− 0.46	(− 1.00, 0.07)	0.089
4 or more NCDs		− 1.01	(− 1.64, − 0.37)	0.002		− 0.59	(− 1.12, − 0.06)	0.029

	*N*	Odds ratio	95% CI	*p*-value	*N*	Odds ratio	95% CI	*p*-value
Tandem stand (< 10 s)	204				209			
1 NCDs		0.44	(0.14, 1.39)	0.164		0.41	(0.14, 1.16)	0.093
2 NCDs		1.15	(0.40, 3.34)	0.792		1.01	(0.40, 2.57)	0.986
3 NCDs		1.72	(0.43, 6.82)	0.439		1.42	(0.46, 4.41)	0.539
4 or more NCDs		5.49	(1.43, 21.01)	0.013		1.19	(0.38, 3.75)	0.767
Low physical functioning score (< = 9)	179				194			
1 NCDs		1.29	(0.55, 3.02)	0.556		1.22	(0.51, 2.93)	0.651
2 NCDs		1.40	(0.56, 3.48)	0.473		1.13	(0.45, 2.85)	0.801
3 NCDs		1.76	(0.50, 6.25)	0.382		5.57	(0.98, 31.59)	0.052
4 or more NCDs		1	(1.00, 1.00)			4.17	(0.82, 21.37)	0.086

Adjusted for age, BMI, smoker status, alcohol consumption and social class

Polypharmacy is common in individuals with multimorbidity; we thus explored whether there were any associations between the number of medications an individual takes and physical functioning (see Table 3). In men there was no significant association between the number of medications taken and all our recorded measures of physical functioning. Conversely, in women there was a significant association between taking 6 or more medications and impaired gait speed (β − 0.61, 95% CI − 1.13, 0.09 *p* = 0.021) and timed up-and-go (β 0.62, 95% CI 0.10, 1.15 *p* = 0.021). We observed similar results when looking at the number of systems medicated (see Supplementary Table 1).

**Table 3:** Number of medications (categorical) as an explanatory variable for physical functioning outcomes, by sex

	Men				Women			
	*N*	Regression coefficient	95% CI	*p*-value	*N*	Regression coefficient	95% CI	*p*-value
Gait speed (FY *z*-score)	196				202			
1–2 medications		− 0.15	(− 0.72, 0.43)	0.612		0.17	(− 0.36, 0.69)	0.532
3–5 medications		− 0.07	(− 0.62, 0.48)	0.804		− 0.48	(− 0.99, 0.04)	0.068
6 or more medications		− 0.39	(− 0.95, 0.17)	0.173		− 0.61	(− 1.13, − 0.09)	0.021
Timed up-and-go (FY *z*-score)	192				200			
1–2 medications		0.10	(− 0.44, 0.63)	0.725		0.07	(− 0.46, 0.61)	0.785
3–5 medications		0.17	(− 0.34, 0.69)	0.509		0.31	(− 0.21, 0.84)	0.240
6 or more medications		0.47	(− 0.06, 0.99)	0.081		0.62	(0.10, 1.15)	0.021
Chair rises (FY *z*-score)	185				185			
1–2 medications		− 0.43	(− 0.97, 0.12)	0.125		− 0.32	(− 0.90, 0.26)	0.271
3–5 medications		− 0.24	(− 0.77, 0.28)	0.360		− 0.10	(− 0.66, 0.46)	0.727
6 or more medications		0.00	(− 0.53, 0.54)	0.989		0.41	(− 0.16, 0.99)	0.156
Physical functioning score (FY *z*-score)	194				194			
1–2 medications		0.15	(− 0.47, 0.76)	0.641		0.19	(− 0.41, 0.80)	0.530
3–5 medications		− 0.01	(− 0.60, 0.57)	0.966		− 0.02	(− 0.61, 0.58)	0.954
6 or more medications		− 0.28	(− 0.88, 0.32)	0.365		− 0.54	(− 1.14, 0.05)	0.075

	*N*	Odds ratio	95% CI	*p*-value	*N*	Odds ratio	95% CI	*p*-value
Tandem stand (< 10 s)	204				209			
1–2 medications		1.98	(0.21, 18.62)	0.552		1.09	(0.19, 6.18)	0.921
3–5 medications		3.56	(0.43, 29.74)	0.242		0.85	(0.15, 4.70)	0.851
6 or more medications		4.51	(0.54, 37.61)	0.164		2.35	(0.45, 12.23)	0.310
Low physical functioning score (< = 9)	194				194			
1–2 medications		0.69	(0.19, 2.46)	0.569		0.62	(0.16, 2.38)	0.483
3–5 medications		1.11	(0.33, 3.70)	0.865		0.91	(0.24, 3.43)	0.894
6 or more medications		1.56	(0.45, 5.40)	0.482		3.58	(0.84, 15.29)	0.085

Adjusted for age, BMI, smoker status, alcohol consumption and social class

## Discussion

The aim of this study was to examine associations between the number of self-reported NCDs, medications and medicated systems with physical function tests in men and women. We found that the proportion of individuals with a low physical functioning score (≤ 9) was high in both sexes, even in a community-dwelling cohort such as ours, but significantly higher in women than men (71.2% and 56.9%, respectively). This is consistent with previous studies that have demonstrated that women have greater prevalence and incidence of mobility disability than men [20, 21]. Our data have demonstrated in both sexes that there is an association between the number of NCDs and physical functioning score with a threshold effect seen at 4 or more self-reported NCDs. This is consistent with previous studies of multimorbidity [22]. Interestingly, in our population, physical functioning was generally not impaired in both sexes unless there were a relatively high number of four NCDs recorded. This is in contrast with the Longitudinal Aging Study Amsterdam, which showed decline in physical functioning was associated with a lower number of chronic diseases (adjusted ORs from 1.58 for 1, to 4.05 for ≥ 3 diseases) [23]. This observation may be partly a reflection of the ‘healthy’ responder bias in the HCS [24], whereby individuals who volunteered to partake in this research have a strong interest in their own health and therefore maintain activity despite multimorbidity. However, almost all the measurements of physical functioning are impaired if four or more NCDs are present.

We also explored how the number of medications and medicated systems impacted upon physical functioning and found an association with gait speed and timed up-and-go tests in women. It has been well established in previous studies that polypharmacy is more common in women than men [25] and our findings support this. Few studies have explored how sex differences influence the impact of polypharmacy on physical function but an association between polypharmacy and impaired physical functioning in women is consistent with previous studies; the Women’s Health Initiative Observational Study (WHI) in the United States showed a risk ratio of incident disability of 1.95 (1.54–2.46) when comparing ≥ 5 with 0–5 medications [26] and an earlier study encompassing community-dwelling women over 65 years of age in Maryland, USA demonstrated an association between impaired physical activities and over-the-counter and prescription medicines [27]. A previous study by Gnjidic and colleagues looking at older men in Sydney, Australia, enrolled in the Concord Health and Aging in Men Project, did demonstrate an association between polypharmacy and physical function in men [28], which we did not observe in our cohort. A possible explanation as to why polypharmacy is associated with poorer physical function in women but not men in our cohort are sex differences in the type and severity of NCDs. A higher proportion of women than men in our cohort reported osteoarthritis (43.4% vs 33.8%) and rheumatoid arthritis (5.0% and 4.5%) which are disorders whose detrimental impact on physical functioning is well documented [29]. Additionally, biological differences between men and women may play a role; it has previously been shown that the sex of the patient can have profound influences on drug metabolism, efficacy and therefore adverse effects [30]. These differences are likely to be secondary to sex-specific differences in body composition (e.g. proportion of body fat) and drug metabolising enzyme (e.g. cytochrome P450) activity [31]. Therefore, it is biologically plausible that the women in our cohort are more susceptible to the detrimental effects of polypharmacy on physical function and this is an important consideration in the clinical setting.

## Limitations and strengths

The number of NCDs was low in both sexes compared with other studies [4, 5] but this may reflect a ‘healthy’ responder bias in the HCS [24] and was equal in men and women. Furthermore, our study population may not be representative of the wider UK population. However, we have previously demonstrated that this cohort is representative of the general population with regard to body build and lifestyle factors, therefore suggesting that selection bias was minimal [24]. As to be expected with all studies of this nature there was some missing data; however, in the current study this was low (ranging from 0 to 13.5% for each variable). This study used a simple question to record NCDs—it did not consider duration or severity of the illness, nor did we attempt to validate it. Furthermore, the questionnaire did not address whether a participant had subsequently fully recovered from a past illness which may therefore no longer adversely affect their physical functioning. Future studies are therefore indicated to address how the severity and duration of illness may impact physical function. However, the specific purpose of the study was to assess whether such analysis might be predictive of low physical function—as such, it could be useful as a simple self-administered screening tool in clinical care.

## Conclusions

In this study, self-report of 4 or more NCDs was associated with an increased risk of poor physical function, an outcome which has previously been associated with adverse clinical sequelae. The study was community based, rather than sited in a hospital outpatient setting, increasing the generalizability of our observations. This observation may be useful for clinicians caring for older adults in a community setting and may lead to the development of a simple screening tool for poor physical functioning.

## Electronic supplementary material

Below is the link to the electronic supplementary material.

Electronic supplementary material 1 (DOCX 16 kb)
