# Supplementary material for: Does self-report of multimorbidity in later life predict impaired physical functioning, and might this be useful in clinical practice?
Source: Aging Clin Exp Res. 2020 Feb 13;32(8):1443–50. doi: 10.1007/s40520-020-01500-8 (PMC7452933; doi:10.1007/s40520-020-01500-8)
Supplement: Supplementary file 1 — Electronic supplementary material 1 (DOCX 16 kb) [file 40520_2020_1500_MOESM1_ESM.docx]

**Supplementary Table 1** Number of systems medicated (categorical) as an explanatory variable for physical functioning outcomes, by sex.^1^

|  | **Men** | | | | **Women** | | | |
| --- | --- | --- | --- | --- | --- | --- | --- | --- |
|  | **N** | **Regression coefficient** | **95% CI** | **p-value** | **N** | **Regression coefficient** | **95% CI** | **p-value** |
| **Gait speed (FY z-score)** | 196 |  |  |  | 202 |  |  |  |
| 1 system |  | -0.17 | (-0.75, 0.41) | 0.563 |  | 0.21 | (-0.35, 0.77) | 0.459 |
| 2 systems |  | -0.15 | (-0.72, 0.42) | 0.601 |  | -0.21 | (-0.74, 0.33) | 0.449 |
| 3 systems |  | -0.10 | (-0.71, 0.52) | 0.752 |  | -0.68 | (-1.22, -0.14) | **0.013** |
| 4 or more systems |  | -0.34 | (-0.91, 0.24) | 0.253 |  | -0.47 | (-1.00, 0.05) | 0.077 |
|  |  |  |  |  |  |  |  |  |
| **Timed up-and-go (FY z-score)** | 192 |  |  |  | 200 |  |  |  |
| 1 system |  | 0.00 | (-0.53, 0.53) | 0.995 |  | -0.01 | (-0.56, 0.55) | 0.979 |
| 2 systems |  | 0.39 | (-0.13, 0.92) | 0.139 |  | 0.13 | (-0.40, 0.66) | 0.632 |
| 3 systems |  | 0.08 | (-0.49, 0.65) | 0.791 |  | 0.51 | (-0.03, 1.04) | 0.063 |
| 4 or more systems |  | 0.43 | (-0.11, 0.96) | 0.120 |  | 0.63 | (0.11, 1.16) | **0.019** |
|  |  |  |  |  |  |  |  |  |
| **Chair rises (FY z-score)** | 185 |  |  |  | 185 |  |  |  |
| 1 system |  | -0.43 | (-0.98, 0.11) | 0.118 |  | -0.54 | (-1.14, 0.06) | 0.077 |
| 2 systems |  | -0.25 | (-0.80, 0.29) | 0.356 |  | -0.04 | (-0.62, 0.53) | 0.886 |
| 3 systems |  | -0.11 | (-0.70, 0.48) | 0.723 |  | 0.03 | (-0.55, 0.62) | 0.907 |
| 4 or more systems |  | 0.02 | (-0.53, 0.57) | 0.946 |  | 0.40 | (-0.17, 0.98) | 0.170 |
|  |  |  |  |  |  |  |  |  |
| **Physical functioning score (FY z-score)** | 194 |  |  |  | 194 |  |  |  |
| 1 system |  | 0.09 | (-0.53, 0.71) | 0.782 |  | 0.31 | (-0.33, 0.95) | 0.342 |
| 2 systems |  | -0.07 | (-0.67, 0.54) | 0.823 |  | -0.01 | (-0.63, 0.61) | 0.970 |
| 3 systems |  | 0.04 | (-0.62, 0.70) | 0.905 |  | -0.26 | (-0.88, 0.36) | 0.413 |
| 4 or more systems |  | -0.24 | (-0.86, 0.38) | 0.448 |  | -0.42 | (-1.04, 0.19) | 0.175 |
|  |  |  |  |  |  |  |  |  |
|  |  |  |  |  |  |  |  |  |
|  | **N** | **Odds ratio** | **95% CI** | **p-value** | **N** | **Odds ratio** | **95% CI** | **p-value** |
| **Tandem stand (<10s)** | 204 |  |  |  | 209 |  |  |  |
| 1 system |  | 2.08 | (0.23, 19.12) | 0.518 |  | 0.40 | (0.06, 2.88) | 0.363 |
| 2 systems |  | 3.93 | (0.46, 33.61) | 0.212 |  | 1.55 | (0.28, 8.52) | 0.616 |
| 3 systems |  | 2.99 | (0.32, 28.20) | 0.338 |  | 1.32 | (0.23, 7.50) | 0.754 |
| 4 or more systems |  | 4.63 | (0.55, 39.31) | 0.160 |  | 2.34 | (0.44, 12.37) | 0.317 |
|  |  |  |  |  |  |  |  |  |
| **Low physical functioning score (<=9)** | 194 |  |  |  | 194 |  |  |  |
| 1 system |  | 1.19 | (0.33, 4.25) | 0.788 |  | 0.47 | (0.11, 1.99) | 0.305 |
| 2 systems |  | 0.85 | (0.24, 2.93) | 0.792 |  | 0.87 | (0.22, 3.47) | 0.840 |
| 3 systems |  | 0.74 | (0.19, 2.91) | 0.667 |  | 1.51 | (0.36, 6.31) | 0.569 |
| 4 or more systems |  | 1.76 | (0.49, 6.36) | 0.387 |  | 2.33 | (0.54, 9.99) | 0.256 |

^1^Adjusted for age, BMI, smoker status, alcohol consumption and social class.

**Supplementary table 2**. List of baseline self-reported NCDs

| **NCDs** | |
| --- | --- |
| High blood pressure |  |
| Diabetes |  |
| Lung disease | e.g. asthma, chronic bronchitis, emphysema, or COPD (chronic obstructive pulmonary disease) |
| Rheumatoid arthritis |  |
| Multiple sclerosis |  |
| Thyroid disease |  |
| Vitiligo |  |
| Depression |  |
| Parkinson’s disease |  |
| Heart disease | e.g. hearth attack, angina, or heart failure |
| Peripheral arterial disease | e.g. claudication |
| Stroke |  |
| Osteoporosis |  |
| Cancer | type of cancer to be specified |
| Any other serious illnesses |  |
